# Bridging Policy and Practice: Reforming Prior Authorization in Kidney Care

**DOI:** 10.34067/KID.0000000657

**Published:** 2024-11-19

**Authors:** Ankur D. Shah, Amy Beckrich, Robert E. Blaser

**Affiliations:** 1 Warren Alpert Medical School of Brown University, Providence, Rhode Island; 2 Division of Kidney Disease and Hypertension, Department of Medicine, Rhode Island Hospital, Providence, Rhode Island; 3 Renal Physicians Association, Rockville, Maryland

**Keywords:** health policy, patient-centered care

Prior authorization (PA) for medications has become an increasingly common and contentious practice in the US health care system. This process, whereby health care professionals must obtain approval from insurance companies before prescribing certain medications, was initially implemented as a cost-containment measure. However, its effect on patient care, health care delivery, and overall system efficiency has sparked ongoing debate among clinicians, payers, policymakers, and patients. In nephrology, where patients often require complex medication regimens and evidence is rapidly evolving, the PA process can have particularly significant implications. This perspective examines the current state of medication PA in nephrology, exploring its intended benefits, unintended consequences, and potential paths forward.

## The Rationale for PA

The primary arguments in favor of PA center on controlling health care costs and promoting evidence-based prescribing practices:

1. Cost containment: By requiring justification for expensive or frequently misused medications, insurers aim to reduce unnecessary spending and encourage the use of more cost-effective alternatives when appropriate.
2. Promoting evidence-based care: PA criteria often align with clinical guidelines, theoretically steering health care professionals toward best practices and discouraging the use of medications for off-label or unproven indications.
3. Patient safety: For medications with significant risks or narrow therapeutic windows, PA can serve as an additional layer of review to prevent inappropriate prescribing.
4. Fraud and abuse prevention: The PA process can help identify and curb prescription drug abuse or diversion.

### Unintended Consequences

1. Administrative burden: The time and resources required to complete PA requests can be substantial. On average, practices complete 45 PAs per week, and physicians and staff spend 14 hours per week on the task.^1^
2. Treatment delays: The PA process can lead to significant delays in initiating or modifying treatment, potentially compromising patient outcomes. Ninety-four percent of physicians report care delays related to PA.^1^
3. Interference with clinical decision making: PA requirements can undermine the physician–patient relationship and interfere with individualized care plans on the basis of clinical judgment and patient-specific factors. A survey of US medical professionals found that PA requirements, especially step therapy, significantly influenced clinical decision making, sometimes leading medical professionals to alter treatment plans to avoid PA burdens.^2^
4. Disparities in care: PA processes may disproportionately affect vulnerable populations, including those with limited health literacy or resources to navigate complex health care systems.^3^ CKD and ESKD disproportionately affect racial and ethnic minorities and individuals of lower socioeconomic status.^4^ PA requirements are likely to compound these existing disparities because these populations face even greater challenges in navigating approval processes.
5. Inconsistent criteria: PA requirements and criteria can vary widely between insurers and even between different plans offered by the same insurer, creating confusion and inefficiency.
6. Limited flexibility: Standard PA criteria may not account for unique clinical situations or patient characteristics, potentially leading to inappropriate denials.

## Impact on Nephrology Practice and Patient Care

The effects of PA are particularly pronounced in nephrology because of the complexity of kidney disease management and the frequent need for specialized medications. Studies have shown that PAs can lead to treatment delays, increased administrative workload, and potential health disparities.^5,6^ In the context of nephrology, where timely access to medications and treatments is often critical, these delays can have serious implications. A recent study demonstrated that in patients subjected to PA, only half prescribed a sodium-glucose cotransporter-2 inhibitors (SGLT2i) received a drug in the class.^6^ Often, the peer making PA decisions for payers is trained in a different specialty than the specialist determining the course of treatment, also raising questions over the face validity of the peer-to-peer process. Most physicians report that health plan peers lack appropriate qualifications to make these determinations.^1^ Nephrologists have trained for years to be able to provide highly specialized and complex care, and peers trained in other specialties making medical decisions for their patients may not be qualified to do so. For instance, the increased probability of emergency dialysis use among patients with ESKD subject to PA for nonemergent ambulance transport is particularly concerning.^7^ Furthermore, the high approval rates for PAs in certain contexts, such as immunosuppressive medications for transplant recipients, call into question the necessity and efficacy of these requirements.^8^ Even when approved, PAs can result in treatment delays and harm. In one study, despite 96% of infusible medications for rheumatologic conditions eventually being approved, the process resulted in treatment delays and greater glucocorticoid exposure.^5^ Traditional Medicare rarely requires PAs because the Centers for Medicare & Medicaid Services (CMS) makes most coverage decisions transparently through established guidelines. Looking forward, the expansion of access to Medicare Advantage plans in patients with kidney disease is likely to result in even greater burden of PA, as by 2019, approximately three in four enrollees were in plans requiring PA.^9^ The cumulative effect of PAs on nephrology practices not only strains resources, but also potentially compromises patient care, highlighting the need for a re-evaluation of these policies.

## Case Example

A 62-year-old woman with stage 3b CKD due to IgA nephropathy presented to her nephrologist for routine follow-up. Her latest laboratory test results showed worsening proteinuria and a slight decline in eGFR. Given the strong evidence supporting SGLT2i for renoprotection in patients with CKD, her nephrologist decided to prescribe an SGLT2i.

However, her insurer required PA for SGLT2i. The nephrologist's office submitted the PA request, including clinical notes, laboratory test results, and justification on the basis of current guidelines. After a week without response, they followed up and were told additional information was needed because there was a step-therapy requirement to fail metformin. The nephrologist requested a peer-to-peer review, noting that the SGLT2i was not being used for diabetes or glycemic control. This process repeated over the next 3 months, with multiple phone calls and faxes exchanged. A follow-up visit was scheduled, reducing access to the nephrologist for their other patients.

In the meantime, the patient's BP increased, and she developed worsening edema. Three months after the initial prescription attempt, the PA was finally approved. However, the delay not only caused unnecessary stress for the patient, but also potentially allowed further progression of her kidney disease during a period when early intervention could have been beneficial.

## Pending Legislation

As of October 2024, the primary legislative initiative to address PA is the Improving Seniors' Timely Access to Care Act of 2024 (S.4532/H.R.8702).^10^ The bill includes provisions that would (*1*) require implementation of an electronic PA system for Medicare Advantage (MA) plans, with standardized processes; (*2*) enhance transparency regarding MA PA requirements; (*3*) delegate explicit authority to the CMS to implement this newly defined real-time PA decision-making process for routinely approved services in MA; and (*4*) delegate explicit authority to the Secretary of Health and Human Services to enforce real-time PA processes for routinely approved services and issue tighter time lines for health plans to make utilization management decisions, such as 24 hours for emergent services. These provisions would apply to all PA reviews, including those for medications.

If this bill is not enacted by the end of 2024, it would have to be reintroduced in the 119th Congress.

## Strategies for Improvement

While eliminating PA entirely may not be feasible or desirable, several strategies could help streamline the process and mitigate its negative impacts:

1. Standardization and automation: Developing standardized electronic PA forms and integrating them into electronic health records could significantly reduce administrative burden and processing times.
2. Gold-carding programs: Implementing programs that exempt health care professionals with consistently high PA approval rates from routine PA requirements could streamline care for many patients.
3. Transparency and communication: Insurers should provide clear, easily accessible information about PA criteria and processes and offer efficient channels for health care professional–payer communication. PA criteria, processes, and appeal procedures should be clearly communicated to patients and health care professionals. Payers should provide detailed explanations for PA denials and guidance on alternative options.
4. Expedited processes: Establishing rapid PA pathways for time-sensitive treatments or for patients with certain high-risk conditions could help prevent dangerous delays in care.
5. Appeals and peer-to-peer reviews: Ensuring efficient, clinically informed appeal processes and timely access to peer-to-peer reviews with appropriate specialists can help resolve complex cases.
6. Flexibility for special populations: Develop mechanisms to accommodate patients with rare diseases, complex comorbidities, or other special circumstances that may not fit standard PA criteria.
7. Outcomes monitoring: Systematically track and analyze the effects of PA policies on patient outcomes, health care utilization, and overall costs to inform continuous improvement efforts.
8. Support legislation: Enact legislative initiatives, such as the Improving Seniors' Timely Access to Care Act, that would mandate use of electronic PA systems, improve transparency, and explicitly define the Department of Health and Human Services/CMS's implementation and enforcement responsibilities, with required reporting on these activities.

## Supplementary Material
