# Supplementary material for: Bridging Policy and Practice: Reforming Prior Authorization in Kidney Care
Source: Kidney360. 2024 Nov 19;6(1):156–8. doi: 10.34067/KID.0000000657 (PMC11793180; doi:10.34067/KID.0000000657)
Supplement: Supplementary file 1 [file kidney360-6-156-s001.pdf]

ASN American Society of Nephrology | JASN Journal of the American Society of Nephrology | KIDNEY360 Accessing Our World From Every Angle | CJASN Clinical Journal of the American Society of Nephrology

ASN Journal Disclosure Form

As per ASN journal policy, I have disclosed any financial relationships or commitments I have held in the past 36 months as included below. I have listed my Current Employer below to indicate there is a relationship requiring disclosure. If no relationship exists, my Current Employer is not listed.

A. Beckrich reports the following:
Employer: Self - Renal Physicians Association (501c6) staff; Spouse - Freddie Mac

I understand that the information above will be published within the journal article, if accepted, and that failure to comply and/or to accurately and completely report the potential financial conflicts of interest could lead to the following: 1) Prior to publication, article rejection, or 2) Post-publication, sanctions ranging from, but not limited to, issuing a correction, reporting the inaccurate information to the authors' institution, banning authors from submitting work to ASN journals for varying lengths of time, and/or retraction of the published work.

Name: Amy Beckrich
Manuscript ID: K360-2024-000849R1
Manuscript Title: Bridging Policy and Practice: Reforming Prior Authorization in Kidney Care
Date of Completion: October 22, 2024
Disclosure Updated Date: October 22, 2024

ASN American Society of Nephrology
JASN Journal of the American Society of Nephrology
KIDNEY360 Accessing Our World From Every Angle
CJASN Clinical Journal of the American Society of Nephrology

ASN Journal Disclosure Form

As per ASN journal policy, I have disclosed any financial relationships or commitments I have held in the past 36 months as included below. I have listed my Current Employer below to indicate there is a relationship requiring disclosure. If no relationship exists, my Current Employer is not listed.

R. Blaser reports the following:
Employer: Renal Physicians Association

I understand that the information above will be published within the journal article, if accepted, and that failure to comply and/or to accurately and completely report the potential financial conflicts of interest could lead to the following: 1) Prior to publication, article rejection, or 2) Post-publication, sanctions ranging from, but not limited to, issuing a correction, reporting the inaccurate information to the authors' institution, banning authors from submitting work to ASN journals for varying lengths of time, and/or retraction of the published work.

Name: Robert Blaser
Manuscript ID: K360-2024-000849R1
Manuscript Title: Bridging Policy and Practice: Reforming Prior Authorization in Kidney Care
Date of Completion: October 23, 2024
Disclosure Updated Date: October 23, 2024

ASN American Society of Nephrology | JASN Journal of the American Society of Nephrology | KIDNEY360 Accessing Our World From Every Angle | CJASN Clinical Journal of the American Society of Nephrology

ASN Journal Disclosure Form

As per ASN journal policy, I have disclosed any financial relationships or commitments I have held in the past 36 months as included below. I have listed my Current Employer below to indicate there is a relationship requiring disclosure. If no relationship exists, my Current Employer is not listed.

A. Shah reports the following:
Employer: Brown Physicians Inc; Consultancy: Otsuka, Calliditas; and Advisory or Leadership Role: American College of Physicians Rhode Island Chapter Governors Advisory Council ; American Society of Nephrology Policy and Advocacy Committee; Renal Physicians Association Government Affairs Committee; Renal Physicians Association Policy Advocacy Leadership Steering Committee.

I understand that the information above will be published within the journal article, if accepted, and that failure to comply and/or to accurately and completely report the potential financial conflicts of interest could lead to the following: 1) Prior to publication, article rejection, or 2) Post-publication, sanctions ranging from, but not limited to, issuing a correction, reporting the inaccurate information to the authors' institution, banning authors from submitting work to ASN journals for varying lengths of time, and/or retraction of the published work.

Name: Ankur Shah
Manuscript ID: K360-2024-000849R1
Manuscript Title: Bridging Policy and Practice: Reforming Prior Authorization in Kidney Care,
Date of Completion: November 5, 2024
Disclosure Updated Date: November 5, 2024
